# A novel mutation in *NCF2* resulting in very-early-onset colitis and juvenile idiopathic arthritis in a patient with chronic granulomatous disease

**DOI:** 10.1186/s13223-019-0386-6

**Published:** 2019-11-21

**Authors:** Suzan AlKhater

**Affiliations:** 1 Department of Pediatrics, College of Medicine, Imam Abdulrahman Bin Faisal University, Dammam, Saudi Arabia; 2 King Fahad Hospital of the University, P.O. Box 2208, Al-Khobar, 31592 Saudi Arabia

**Keywords:** Chronic granulomatous disease, Primary immunodeficiency, Early-onset colitis, Inflammatory bowel disease, Juvenile idiopathic arthritis

## Abstract

**Background:**

Chronic granulomatous disease (CGD) is a rare primary immunodeficiency disorder caused by a defect in the nicotinamide adenine dinucleotide phosphate (NADPH) oxidase complex. The disease primarily presents with recurrent infections, and patients may also present with inflammatory conditions, including noninfectious colitis, and an increased frequency of autoimmunity. We report here a patient with CGD in whom the presentation, unlike the classical presentation of CGD, was predominantly of an inflammatory and autoimmune phenotype.

**Case presentation:**

A 3-year-old Pakistani female presented with bloody diarrhea since the age of 7 days, followed by the development of perianal abscesses and fistula. There was no other history of recurrent infections. The patient subsequently developed joint pain and stiffness with persistently elevated inflammatory markers and elevated anti-cyclic citrullinate peptide (anti-CCP) antibody titer. She was diagnosed with oligoarticular juvenile idiopathic arthritis and colitis. The diagnosis of CGD was later made and was based on the absence of NADPH oxidase activity in the patient’s neutrophils upon phorbol myristate acetate (PMA) stimulation using the dihydrorhodamine-1,2,3 (DHR) flow cytometry test. Targeted next-generation sequencing revealed an unreported deletion mutation in exon 10 as a homozygous loss-of-function variant of the human neutrophil oxidase factor 2 (*NCF2*) (*NCF2*: NM_001190789, nucleotide change: c.855_856del:p.T285fs). The gene encodes a protein subunit, p67^phox^, in the NADPH enzyme complex.

**Conclusions:**

The case emphasizes the importance of maintaining high clinical suspicion of immunodeficiency and CGD in patients with very-early-onset colitis and autoimmune disorders. This case is important due to its rarity and because it might represent a previously undiscovered mutation, which is possibly more common in the patient’s ethnic group. Other mutations in *NCF2* have been linked to inflammatory bowel disease and autoimmunity, but without CGD, suggesting similarities in the pathogenesis.

## Background

Primary immunodeficiency disorders (PIDs) are an inherited group of disorders characterized by a unique combination of the inability to fight pathogens, resulting in recurrent infections, and immune dysregulation causing autoimmune diseases [1, 2].

Chronic granulomatous disease (CGD) is a rare PID with an incidence of 1:250,000 live births in Western countries [2]. The disease is usually severe and diagnosed in infancy, with the only curative treatment being hematopoietic stem cell transplantation, although gene therapy and gene editing are promising alternatives [3, 4].

CGD is caused by a defect in phagocytic neutrophils that inhibits the capability of eliminating intracellular pathogens [2]. This is caused by mutations that affect the nicotinamide adenine dinucleotide phosphate (NADPH) oxidase complex, a transmembrane protein that clears pathogens by generating reactive oxygen species (ROS) [2]. X-linked mutations (XL-CGD), are limited mostly to males and are located on the *CYBB* gene that codes for gp91-phox protein [4]. The XL form of CGD account for approximately two-thirds of cases of in Westerners [5]. The mutations that cause autosomal recessive CGD (AR-CGD) encode the components of the phagocytic NADPH oxidase complex—p22^phox^, p47^phox^, p67^phox^ p40^phox^, and EROS subunits—and are located in genes *CYBA, NCF1, NCF2, NCF4* and *CYBC1*, respectively [5, 6]. The AR forms of CGD are more commonly reported in studies from Turkey [7], Israel [8], Arab countries and North Africa [9], Iran [10], and India [11], presumably because of the higher rate of consanguineous marriage (Table 1).

**Table 1:** Summary of X-linked and autosomal recessive forms of chronic granulomatous disease genes according to ethnic origin

Publication [reference]	Number and population	X-linked^a^	Autosomal recessive^b^	Type of autosomal recessive gene
Köker et al. [7]	89 Turkish	34 (38.2%)	50 (56.2)	*CYBA* (22.5%) *NCF1* (19.1%) *NCF2* (14.6%)
Van den Berg et al. [9]	357 European	265 (74.2%)	92 (25.7%)	*NCF1* (16%) *CYBA* (5.1%) *NCF2* (2.5%) Unknown (8.6%)
	12 Turkey^c^	4 (33%)	8 (67%)	
	38 Arab/North African^c^	11 (29%)	27 (71%)	
	6 East and South Asia	1 (17%)	5 (83%)	
	16 Israeli/Jew	9 (56%)	7 (44%)	
Wolach et al. [8]	84 Israeli (Jew, Arab^c^, visitor)	32 (38%)	52 (62%)^c^ (64% consanguinity)	*NCF1* (31%) *NCF2* (19%) *CYBA* (12%)
Fattahi et al. [10]	93 Iranian^c^	12 (12.9%)	81 (87.1%)	*NCF1* (55.5%)
Kulkarni et al. [11]	90 Indian^c^ (32% consanguinity)	27 (30%)	63 (70%)	*NCF1* (56%) *CYBA* (7%) *NCF1* (7%)

^a^*CYBB*, ^b^*NCF1*, *CYBA*, *NCF2*, *NCF4*

^c^Reported high rate of consanguinity

Of all the mutations associated with CGD, mutations in the *NCF2* gene that affect p67^phox^ expression (p67-^phox^) are the rarest and are previously reported as clinically milder than others [9]. In Western populations, approximately 6% of CGD cases are caused by defects in p67^phox^ [5], a 526-amino acid protein encoded by *NCF2* (gene locus 1q25) [https://www.omim.org/entry/608515].

In addition to susceptibility to recurrent bacterial and fungal infections, inflammatory manifestations are common in CGD. An increased overall risk of developing malignancies has previously been reported in CGD patients [12]. However, this was not supported by findings from recent large cohorts studies [7, 9]. The sporadic cases reported may not present a true cause-effect relationship [9, 13]. On the other hand, among the inflammatory manifestations of CGD, intestinal involvement in the form of diffuse hyperinflammation is the commonest, in both XL and AR forms, and may reach a prevalence of 50% [14]. Patients with CGD appear to be at an elevated risk of developing colitis that is similar to Crohn’s type of inflammatory bowel disease (IBD). In fact, severe intestinal inflammation may be the first clinical sign of CGD, as observed in patients with very-early-onset disease (defined as onset at younger than 6 years of age) [15]. CGD colitis is histologically indistinguishable from classical Crohn’s disease and presents with similar signs, including growth failure, anemia and failure to thrive, abdominal pain, diarrhea, nausea, vomiting and constipation [15]. In addition, *NCF2* mutations that allow for some production of ROS have been associated with susceptibility to autoimmune diseases, including systemic lupus erythematosus (SLE) [16, 17] and sarcoidosis [18].

We present a case in which a patient with early-onset colitis was later diagnosed with CGD and developed symptoms of arthritis. The constellation of disease in our patient is suggestive of another novel mutation in one of the genes that encodes the NADPH oxidase complex that predisposes patients to severe inflammation and autoimmunity.

## Case presentation

Our patient is a 3-year-old Pakistani female born to first-degree cousins. She presented at the age of 10 months with enlarged cervical lymph nodes and diarrhea. She had multiple admissions for recurrent diarrheal illnesses. The diarrhea started at the age of 7 days, with a frequency of more than 10 times per day of watery consistency but no blood or mucus. She was started on an elemental amino acid-based formula because of a diagnosis of food protein-induced enterocolitis syndrome based on a presentation of severe bloody diarrhea, acidosis and hypotension. She had a sister who had a similar presentation of chronic diarrhea and fever and died at the age of 1 year due to septic shock. Immune deficiency was not suspected and the underlying cause was not investigated. Also, no genetic tests were obtained and the family did not receive any prenatal genetic counseling.

After removing cow’s milk from our patient’s diet and initiating the special formula, her diarrhea settled, but at the age of 1 year, she had a recurrence of diarrhea described as bloody and associated with multiple perianal abscesses. The abscesses were treated with intravenous antibiotics and surgically drained. The culture from the drained pus repeatedly grew extended spectrum β-lactamase-producing *E. coli* and *Klebsiella* species. At the age of 2 years, she developed arthritis involving large joints of the lower limb (knees and ankle) and wrist associated with morning stiffness and an inability to walk.

**Fig. 1:**
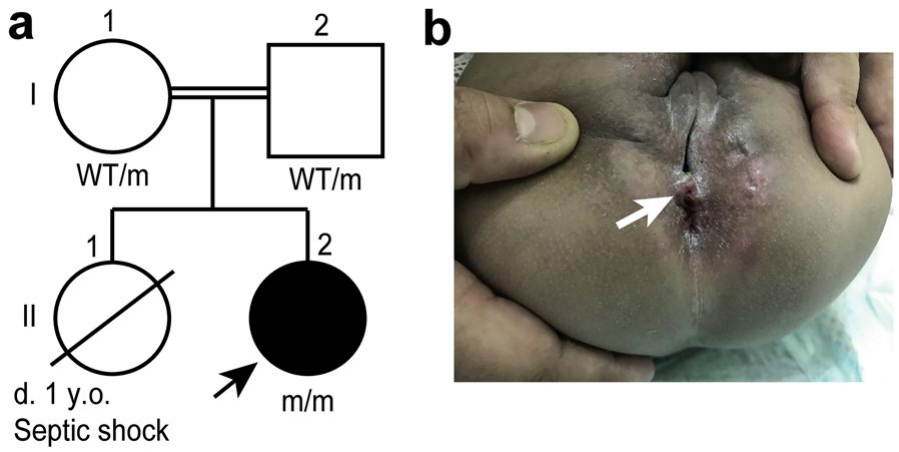
Deficiency of p67^phox^ in a child with AR CGD. **a** Family pedigree. The arrow indicates the proband. Each generation is designated by a Roman numeral (I, II). Blackened symbols denote the affected family members. *m* mutation, *WT* wild type. **b** Scars of previous surgically drained perianal abscesses and a perianal fistula opening at the 12 o’clock position (arrow)

On examination, the patient had severe failure to thrive. Multiple enlarged cervical lymph nodes were noted. The scar of the Bacillus Calmette-Guérin (BCG) vaccine was normal, and no axillary lymph nodes were detected. The liver was palpable 3 cm below the costal margins. She had swelling of the left knee joints with a reduced range of movement. Perineal examination revealed inflammation, multiple scars of drained perianal abscesses and a perianal fistula opening (Fig. 1).

Laboratory investigation revealed leukocytosis (29.1 × 10^9^/L), anemia (hemoglobin 8.4 g/dL), elevated inflammatory markers (C-reactive protein 12.0 mg/dL, and ESR 80 mm/h) and elevated immunoglobulin levels. A rheumatological workup was positive for serological markers of autoimmunity, including antinuclear antibodies (ANA) (titer 1:80, speckled pattern), rheumatoid factor (25.1 IU/mL; normal < 15), anti-cyclic citrullinate peptide (anti-CCP) antibody titer (216.7 u/mL; normal < 20) and anti-double-stranded DNA (anti-dsDNA) antibodies (83.6 IU/mL; normal < 35). In addition, these markers were positive on repeated testing. Other markers, including anti-RNP, anti-SSA, anti-SSB, and anti-Sm, remained negative, while C3 and C4 complement levels were not suppressed. Joint fluid sampling was obtained, and an infectious etiology for joint involvement was excluded by culture obtained from the knee joint. Based on the clinical presentation and laboratory workup, a diagnosis of oligoarticular juvenile idiopathic arthritis (JIA) was provided.

Furthermore, microbiological testing for infectious diarrhea, including stool culture and sensitivity tests, a stool ova and parasite examination, *Clostridium difficile* toxin test, and *Giardia lamblia* and *Cryptosporidium* stool antigen tests, were negative. The celiac panel was also negative, and malabsorption tests, including fecal fat and fecal elastase tests, were normal. Stool examination was positive for fecal occult blood, and fecal calprotectin, an indicator of intestinal inflammation, was elevated at 580 mcg/g (normal < 50.0). Workup for TB was negative, including bone marrow and lymph node examination, using polymerase chain reaction (PCR) assay, cultures, and the TB gold test. In addition, a purified protein derivative (PPD) skin test was performed on the patient and showed no reaction.

**Fig. 2:**
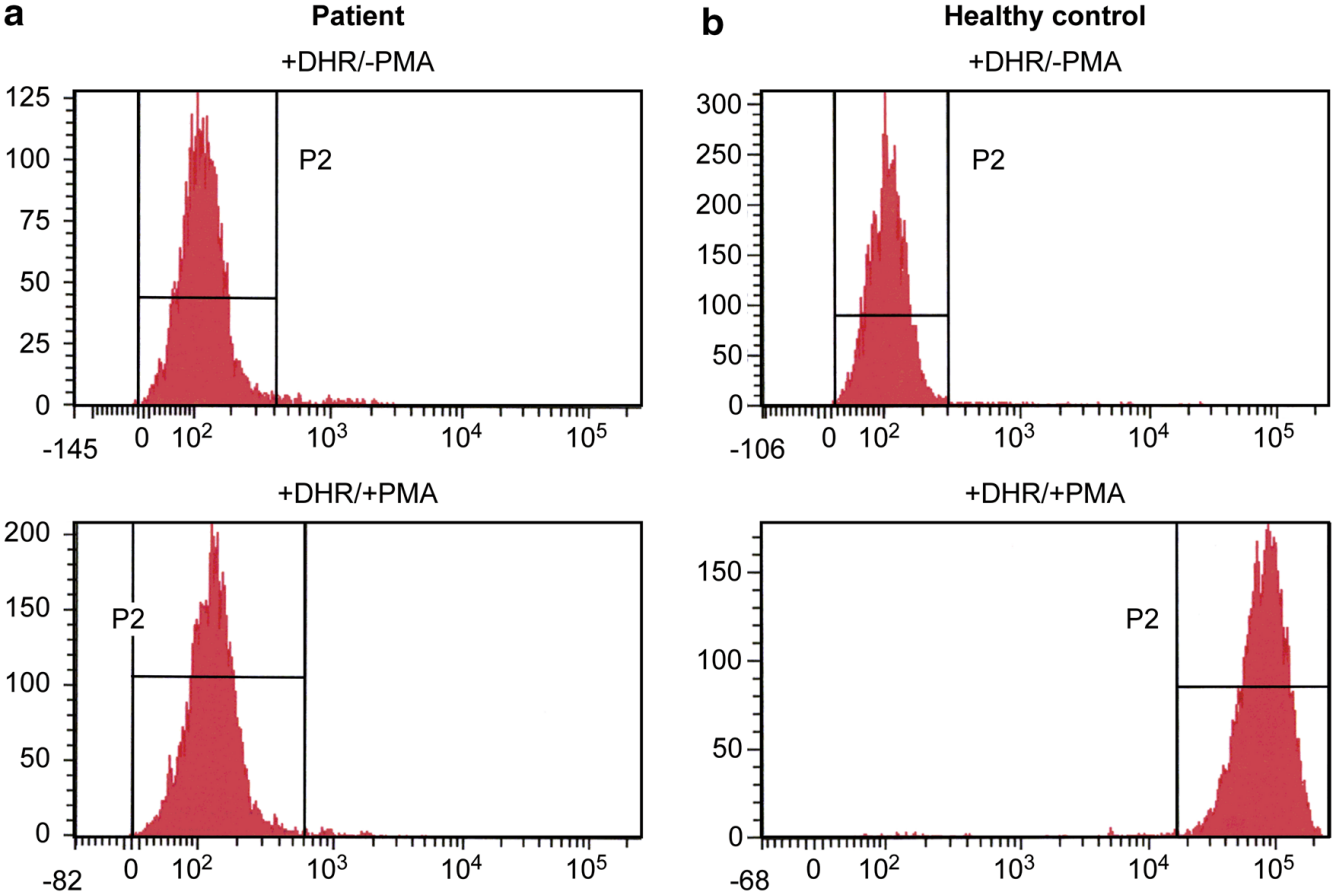
Neutrophil oxidative burst test using DHR. The top graphs represent the unstimulated cells, while the bottom graphs are cells stimulated using PMA. **a** The patient showed an absence of reactivity after PMA stimulation, consistent with CGD. **b** Normal neutrophil respiratory burst showing a complete shift in fluorescence after stimulation in a healthy control. *DHR* dihydrorhodamine-1,2,3; *PMA* phorbol myristate acetate; *CGD* chronic granulomatous disease

In view of her presentation and the early colitis symptoms, she was further evaluated, and a diagnosis of CGD was made based on the oxidative burst assay [dihydrorhodamine-1,2,3 (DHR) flow cytometry test], which revealed an absence of NADPH oxidase activity in the patient’s neutrophils upon phorbol myristate acetate (PMA) stimulation (Fig. 2). The neutrophil oxidative index (NOI) of the patient was < 5. NOI measures the oxidative ability of phagocytes and their overall integrity and is calculated from the ratio of the fluorescence in stimulated phagocytic cells to the fluorescence expressed in unstimulated phagocytic cells [19]. The normal value in control specimens is usually > 73 [19].

The diagnosis of CGD was confirmed by targeted next-generation sequencing using Ion AmpliSeq Designer software (Life Technologies, Carlsbad, CA, USA). The gene panel used analyzed over 200 genes associated with immune deficiency and immune dysregulation, including auto-Inflammatory and Autoimmunity syndromes. The test was performed on venous blood samples collected in EDTA tubes for DNA extraction after obtaining written consent from the parents to perform the genetic diagnostic assay. The test revealed a deletion mutation in exon 10 as a homozygous loss-of-function variant of *NCF2* (*NCF2*: NM_001190789, nucleotide change: c.855_856del:p.T285fs). Both parents were heterozygous for the mutation. We believe this to be a novel variant that has not been previously reported.

To prevent serious and life-threatening infections, the patient was started on an antibacterial and antifungal prophylactic regimen consisting of trimethoprim-sulfamethoxazole (trimethoprim 6 mg/kg/day) and itraconazole (5 mg/kg/day), respectively. She remained free of infection. Her arthritis symptoms were initially managed with naproxen followed by oral prednisolone at a dose of 1 mg/kg/day for 4 weeks followed by gradual tapering and then discontinued after 4 months. In addition, hydroxychloroquine was initiated and continued throughout the observation period, which lasted for 18 months following the onset of arthritis. She showed improvement and started to walk with no pain and had no relapse of symptoms during subsequent follow-up visits. However, we failed to establish long-term follow-up. In addition, further endoscopic, histological and imaging studies were planned but not performed because the patient and family traveled to their home country and were lost to follow-up.

## Discussion and conclusions

We report a case of CGD with a rare presentation of very-early-onset intestinal inflammation and an autoimmune disorder, JIA. The diagnosis of oligoarticular JIA was made according to the widely used International League of Associations for Rheumatology Classification [20], which defines JIA as the presence of arthritis in a child under 16 years of age and lasting at least 6 weeks. Oligoarticular JIA involves fewer than 5 joints during the first 6 months of disease. Our patient was positive for autoimmune antibodies, including ANA, commonly seen in oligoarticular JIA. In addition, tests for other autoantibodies were positive, including that for anti-dsDNA, despite the absence of manifestation of other connective tissue disorders, including lupus. JIA is associated with the formation of various autoantibodies and the development of other connective tissue diseases [21].

Notably, apart from perianal abscesses, which is likely a presentation of CGD colitis, history of other recurrent infections were unreported in our patient. Nevertheless, the pattern of organisms isolated in the patient points to a defective phagocytic component of the immune system, which results in a predisposition to gram-negative organisms, particularly *E. coli* and *Klebsiella* [1]. Furthermore, the early symptoms soon after birth in our patient were likely signs of CGD with intestinal inflammation. The presenting symptoms can be mistaken for food hypersensitivity reactions.

The case is important due to its rarity and because it might represent a previously undiscovered mutation. In addition, it is interesting that the mutation may be more common in the patient’s ethnic group, as other mutations in the *NCF2* gene have been reported in cases from the Middle East and South Asia but not in Western populations.

Similar to our case, Vignesh et al. [22] described three patients from India with a diagnosis of CGD due to *NCF2* defects. All 3 patients had signs of colitis from early infancy, while in two of the three cases, colitis was an initial presenting feature of the disease [22]. One child, a 2-year-old boy, who was ill since birth and developed fulminant colitis at 1½ years, had a homozygous deletion in NCF2 (c835_836delAC:p. T279fsX294), which resulted in a stop codon in exon 10 and reduced expression of the p67^phox^ protein. In addition, all the reported cases had failure to thrive, pneumonia and granulomas. Similar presentations have been reported in Iranian [23] and Turkish children [7] with CGD due to *NCF2* defects.

CGD associated colitis was reported in patients with CGD since the disease was first described. However, specific mutations that are associated with the development of colitis have been reported only recently in CGD patients [7]. Köker et al. [7] noted that patients with the p67^phox^ phenotype had an earlier clinical presentation and were diagnosed at a younger age. Marciano et al. [15] noted GI involvement in 46 of 140 CGD patients (33%), which was more common in XL-CGD. Magnani et al. [24] described the intestinal manifestations of CGD in a cohort of 98 patients; 70% of the cohort had inflammatory episodes, and half of the patients had AR-CGD.

The similarities between Crohn’s disease and CGD patients presenting with colitis may lead to misdiagnosis. Interestingly, mutations affecting the NADPH oxidase complex that involve early-onset colitis, but without CGD, are reported in the literature. Single nucleotide polymorphisms (SNPs) associated with an increased risk of IBD are also located in the genes that encode the NADPH oxidase complex [25]. For many of these mutations, the effect on ROS formation may be insufficient to cause CGD, but the clinical and pathological features of colitis in CGD and Crohn’s disease suggest similarities in pathogenesis [26–28].

Interestingly, a meta-analysis of genome-wide association studies revealed that many loci for susceptibility to IBD overlap with those of other inflammatory and immune-mediated diseases, including PIDs [29]. In fact, many monogenic disorders of the immune system can manifest with immune dysregulation, resulting in an aberrant immune system that is unable to fight pathogens, yet fights against self-tissue, causing autoimmunity [2, 30]. Therefore, although viewed as paradoxical in the past, the combination of immunodeficiency and autoimmune disorders is now understood to be interrelated, with common mechanisms that might be considered “two sides of the same coin” [31].

Indeed, sporadic cases of CGD presenting with autoimmune disorders appeared in the literature. Chou et al. [16] described a child with SLE (a child of consanguineous parents of Arab origin) who had chronic diarrhea since the age of 3 months and later developed failure to thrive, chronic arthralgias, and stiffness in multiple joints. The symptoms of inflammation and autoimmunity delayed a diagnosis of CGD, which was revealed after genetic testing that showed mutations in the *NCF2* gene. In addition, JIA was recently reported in an Iranian child with CGD [32].

Equally important to the IBD gene studies was the identification of specific mutations in *NCF2* that have been considered risk factors for SLE, without an associated immune deficiency or CGD manifestation [33–36]. Although no similar genetic links were reported in rheumatoid arthritis (RA), a link was recently identified between NCF2 and RA by studying the interaction of the various subunits of the neutrophil’s NADPH oxidase complex during its assembly process. Aberrant neutrophils were found to be the disease-causing agent in RA [37]. Defects in NADPH oxidases in neutrophils were found to result in the citrullination of specific proteins, including the citrullination of type II collagen, resulting in the autoantibody formation and autoimmunity observed in RA [37–39]. Moreover, the citrullination of p67^phox^/NCF2 result in the inability to further assemble the NADPH oxidase complex, therefore disrupting the synthesis of ROS [37]. ROS are known for their anti-inflammatory properties and have been reviewed elsewhere [40]; their deficiency has been implicated in the pathogenesis of inflammatory autoimmune diseases.

Although these mechanisms may operate differently in CGD patients, the combination of colitis and RA, or JIA, in a CGD patient, as seen here, suggests that *NCF2* has immunomodulating effects. As a result of failure to assemble the protein subunits of the NADPH complex in CGD and deficient ROS production, the immune system is unable to eradicate pathogens while being at high risk for autoimmunity.

In conclusion, our case illustrates the different facets of PIDs and demonstrates that their presentation is not limited to a predisposition to repeated infections. The predominance of inflammatory manifestations in our patient emphasizes the importance of correctly recognizing the different symptoms and various presentations of PIDs and not overlooking the possibility of complex involvement of intestinal inflammatory manifestations and autoimmunity in patients with CGD. The discovery of more cases of CGD associated with the *NCF2* mutation will be necessary to further characterize this mutation and understand the complex phenotypic associations.

## Data Availability

Any additional data about the materials and methods will be provided by the corresponding authors upon request.

## Abbreviations

JIA: oligoarticular juvenile idiopathic arthritis
BCG: Bacillus Calmette-Guérin
PCR: polymerase chain reaction
PPD: purified protein derivative
DHR: dihydrorhodamine-1,2,3
PMA: phorbol myristate acetate
NOI: neutrophil oxidative index
NADPH: nicotinamide adenine dinucleotide phosphate
CRP: C reactive protein
NCF2: human neutrophil oxidase factor 2
CGD: chronic granulomatous disease
AR-CGD: autosomal recessive
XL-CGD: X-linked
SNPs: single nucleotide polymorphisms
IBD: inflammatory bowel disease
PIDs: primary immunodeficiency disorders
SLE: systemic lupus erythematosus
ANA: antinuclear antibodies
anti-dsDNA: anti-double-stranded DNA
RA: rheumatoid arthritis
CCP: cyclic citrullinate peptide
